# Development and Characterization of PEGylated Poly D,L-Lactic Acid Nanoparticles for Skin Rejuvenation

**DOI:** 10.3390/nano15060470

**Published:** 2025-03-20

**Authors:** Seunghwa Lee, Hyoung-Wook Moon, Seong-Jin Lee, Jin-Cheol Cho

**Affiliations:** R&D Center, CHA Meditech Co., Ltd., 119 Techno 2-ro (#206, Migun Techno World, Yongsan-Dong), Yuseong-gu, Daejeon 34116, Republic of Korea; lshlsh0106@chamc.co.kr (S.L.); rsnom@naver.com (H.-W.M.); sjlee@chamc.co.kr (S.-J.L.)

**Keywords:** PEGylation, PDLLA, collagen, in vivo, skin rejuvenation

## Abstract

Recently, various biocompatible and biodegradable materials have garnered significant attention as cosmetic fillers for skin rejuvenation. Among these, poly ε-caprolactone (PCL), poly L-lactic acid (PLLA), poly D,L-lactic acid (PDLLA), and polydioxanone (PDO) microspheres have been developed and commercialized as a dermal filler. However, its irregularly hydrophobic microspheres pose hydration challenges, often causing syringe needle blockages and side effects such as delayed onset nodules and papules after the procedure. In this study, we synthesized a polyethylene glycol-poly D,L-lactic acid (mPEG-PDLLA) copolymer to address the limitations of conventional polymer fillers. Comprehensive characterization of the copolymer was performed using nuclear magnetic resonance spectroscopy, Fourier transform infrared spectroscopy, and differential scanning calorimetry. The mPEG-PDLLA copolymers demonstrated a unimodal size distribution of approximately 121 ± 20 nm in an aqueous solution. The in vitro cytotoxicity and collagen genesis of mPEG-PDLLA copolymers were evaluated using human dermal fibroblast cells. In this study, angiogenesis was observed over time in hairless mice injected with mPEG-PDLLA copolymers, confirming its potential role in enhancing collagen synthesis. To assess the inflammatory response, the expression levels of the genes MMP1 and IL-1β were analyzed. Additionally, gene expression levels such as transforming growth factor-β and collagen types I and III were compared with Rejuran^®^ in animal studies. The newly developed collagen-stimulating PEGylated PDLLA may be a safe and effective option for skin rejuvenation.

## 1. Introduction

The advancement of medical technology has significantly increased the average human lifespan, contributing to substantial growth in the healthcare market [1,2,3,4]. With the expansion of this market, consumers are increasingly interested not only in skincare products but also in aesthetic procedures that offer safe and long-lasting results. Among various aesthetic procedures, cosmetic fillers represent the most popular category [5,6,7], with hyaluronic acid (HA) fillers being the most widely utilized [8,9,10]. HA is an FDA-approved material recognized for its safety and biodegradability, as it can be enzymatically degraded by hyaluronidase [11,12]. However, the short half-life of HA (1–3 days) significantly limits its applicability as a dermal filler [13]. To address this limitation, cross-linking agents such as 1,4-butanediol diglycidyl ether (BDDE) are commonly used to extend the half-life [14,15,16]. While the efficacy of cross-linked HA fillers has been well established, the presence of residual BDDE may lead to adverse effects, including swelling and inflammation [17]. To mitigate these shortcomings, it is imperative to develop polymer-based fillers that minimize side effects while ensuring high performance and biocompatibility.

A lot of products utilizing biocompatible polymers with excellent safety and minimal side effects have already been developed in the aesthetic filler market. Various polymers are used in dermal fillers, including HA, polyglycolic acid (PGA), poly ε-caprolactone (PCL), and polylactic acid (PLA) [18,19]. Among these, PLA is categorized into poly L-lactic acid (PLLA), poly D,L-lactic acid (PDLLA), and polydioxanone (PDO) based on their molecular arrangement [18]. Both PLLA and PDLLA are considered safe for human application, as they degrade into lactic acid, carbon dioxide (CO_2_), and water upon injection into the body [20]. Sculptra^®^, the most widely used PLLA filler, is designed to promote collagen synthesis through an inflammatory response initiated by macrophage phagocytosis during the degradation process [18,21,22]. The degradation period of Sculptra^®^ is approximately 24 months, during which collagen is progressively regenerated, resulting in a natural enhancement of the skin. These products, often referred to as “skin boosters,” have recently gained significant attention in the aesthetics industry due to their long-lasting effects [23].

PLLA is semi-crystalline and has a regular chain structure, while PDLLA is amorphous, exhibiting an irregular chain structure with a random distribution of D-form and L-form. As a result, PDLLA degrades at a faster rate compared to PLLA, while also exhibiting a reduced incidence of nodule formation [24]. This advantageous property has led to a growing demand for PDLLA skin boosters. In contrast, PDLLA is a hydrophobic polymer, presenting challenges in its dispersion in normal saline. Furthermore, its incomplete dissolution can result in syringe needle blockages during medical procedures. While this issue can be mitigated by using larger needle gauges, such an approach may increase patient discomfort, making it a suboptimal solution.

PEGylation represents a promising strategy to address these challenges. This technique involves the chemical conjugation of PEG to hydrophobic polymers such as PDLLA, thereby enhancing their interaction with water molecules. Through this modification, PEGylation improves the hydrophilicity of polymers, enhances their stability in biological environments, and facilitates practical applications [25,26,27]. In combination with other polymers, it significantly increases dispersion and effectively mitigates problems such as aggregate and blockage. Previous studies have demonstrated that PEGylated polymers exhibit not only improved solubility in aqueous phase but also a reduction in immune responses in vivo [28,29,30]. In addition, the biocompatibility and safety of PEG have been confirmed through subcutaneous injection studies, demonstrating minimal immune response and tissue irritation [31]. Utilizing these properties of PEGylated PDLLA offers an opportunity to address the inherent limitations of PDLLA, thereby enhancing its stability and safety for applications as a dermal filler in cosmetic procedures.

Polynucleotide (PN) has grown significantly in global aesthetics and cosmetics due to its exceptional biocompatibility. PN is produced from the testicles, while PDRN is produced from sperm cells [23]. Rejuran^®^ was the first PN used as a skin booster in 2014. Rejuran^®^ is a widely used skin treatment containing purified salmon DNA fragments, also known as PN, which has been clinically proven to promote tissue regeneration and collagen synthesis [32]. It aids in skin repair and healing, reduces scars, enhances skin texture, and diminishes fine lines and wrinkles.

In this study, Rejuran^®^ was selected as the control due to its similarity to mPEG-PDLLA copolymers in terms of biodegradation period, mechanism of action, and collagen regeneration effects. Therefore, the mPEG-PDLLA copolymers were synthesized to address the limitations associated with PDLLA-based skin boosters, and their physicochemical properties were thoroughly characterized. In addition, biocompatibility assessments have consistently demonstrated the safety and collagen synthesis capability of mPEG-PDLLA copolymers.

## 2. Materials and Methods

### 2.1. Materials

D,L-lactide, fetal bovine serum (FBS), and trypsin-EDTA were purchased from Thermo Fisher Scientific, Inc. (Waltham, MA, USA). Polyethylene glycol monomethyl ether 2000 (mPEG) was obtained from Tokyo Chemical Industry Co., Ltd. (Toshiba, Kita-ku, Tokyo, Japan). Tin (II) 2-ethylhexanoate (SnOct_2_), deuterated chloroform (CDCl_3_), agarose, trypan blue, and neutral red were sourced from Sigma-Aldrich (St. Louis, MO, USA). Ethyl ether was acquired from Samchun Pure Chemical Co., Ltd. (Pyeongtaek, Republic of Korea). Tetrahydrofuran (THF) was purchased from Acros Organics BVBA (Geel, Belgium). A 15 L glass reactor was purchased from Buchi (Uster, Switzerland). The minimum essential medium (MEM), Dulbecco’s phosphate-buffered saline (DPBS), and sodium bicarbonate (NaHCO_3_) were sourced from Himedia (Mumbai, India). Pen–strep solution was purchased from MP Biomedicals (Santa Ana, CA, USA). HDF cells were obtained from Lonza (Basel, Switzerland). Rejuran^®^ was purchased from Pharmaresearch Inc. (Gangneung, Republic of Korea).

### 2.2. Preparation of mPEG-PDLLA Copolymers

**Figure 1:**
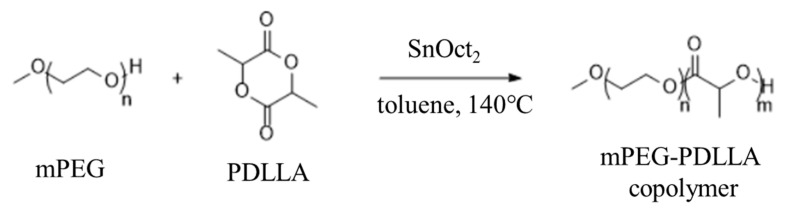
Synthesis of mPEG-PDLLA.

The mPEG-PDLLA copolymers were prepared by the ring-opening reaction of D,L-lactide in the presence of mPEG using SnOct_2_ as a catalyst (Figure 1). mPEG-PDLLA copolymers with various molecular weights were synthesized as follows: in brief, 4 L of toluene and 384.6 g of mPEG (192.1 mmol) were added to the reactor and the temperature was raised to 60 °C to ensure complete dissolution. After releasing the pressure under a nitrogen (N_2_) gas atmosphere, 867.56 g of D,L-lactide (6019.23 mmol) was added by stirring. An amount of 7.70 g of SnOct_2_ (19.23 mmol) was added and the mixture was stirred at 140 °C for 24 h. Following the reaction, the temperature was lowered to 60 °C, and the mixture was condensed. The solution was then slowly added dropwise into the ether at 0 °C with stirring. After stirring for 30 min, the precipitate was collected via filtration. The resulting product was washed twice with ether and dried under vacuum for 3 days. The full list of the resulting products is presented in Table 1.

### 2.3. Analysis of Physicochemical Properties of mPEG-PDLLA

#### 2.3.1. ^1^H-Nuclear Magnetic Resonance and Fourier Transform Infrared

The ^1^H-NMR spectrum was acquired in CDCl_3_ at 24 °C using an NMR spectrometer (BRUKER Fourier 300, BRUKER, Billerica, MA, USA) to characterize the chemical composition of the mPEG-PDLLA copolymers. FT-IR (PerkinElmer Spectrum 2, PerkinElmer, Waltham, MA, USA) was employed to analyze the chemical structure and functional groups of mPEG-PDLLA copolymers. The FT-IR spectrum was obtained by 16 scans at a resolution of 8 cm^−1^.

#### 2.3.2. Gel Permeation Chromatography and Differential Scanning Calorimeter

The average molecular weight (M_w_) of the mPEG-PDLLA copolymers was determined using a GPC system (Waters ACQUITY APC system, Waters, Milford, MA, USA) with four Waters Styragel columns (HR-0.5, 1, 4E, and 4). Samples were dissolved in THF at a concentration of 2 mg/mL and the elution process was carried out at a flow rate of 0.6 mL/min. Calibration of M_w_ was conducted using polystyrene (PS) as the standard. Thermal properties of the mPEG-PDLLA copolymers were obtained using a DSC (DSC1, Mettler-Toledo, Columbus, OH, USA) applying a heating rate of 10 °C/min.

#### 2.3.3. Nano Particle Size Analyzer

The size and distribution of the mPEG-PDLLA copolymers were determined using a nanoparticle size analyzer (NANOPHOX, Sympatec, GmbH, Clausthal-Zellerfeld, Germany). All measurements were performed at 25 °C.

### 2.4. In Vitro Test

#### 2.4.1. Cytotoxicity Assay

The HDF cells were cultured in Dulbecco’s Modified Eagle’s Medium (DEME) supplemented with 10% FBS and 1% penicillin/streptomycin. The cells were cultured in a 5% CO_2_ incubator at 37 °C. HDF cells were seeded at a density of 1 × 10^4^ cells per well in 48-well plates and incubated for 24 h. After this incubation, the culture medium from each well was collected for collagen production tests. Subsequently, 200 µL of fresh medium was added to each well, followed by 20 µL of EZ-Cytox (EZ-BULK150, DoGenBio, Seoul, Republic of Korea) solution. After an additional incubation for 4 h, the absorbance was measured by a microplate reader (INNO-M, LTEK, Seongnam, Republic of Korea) at 450 nm.

#### 2.4.2. Collagen Synthesis Assay

The amount of newly synthesized collagen was measured by Human Pro-Collagen I alpha 1 DuoSet ELISA (DY6220-05, R&D Systems, Minneapolis, MN, USA). A plate of 96 wells was coated with the Pro-Collagen I alpha 1 capture antibody, followed by the addition of 100 µL of either the culture sample, which was incubated overnight at room temperature (mPEG, P1, and P2), or a standard (human Pro-Collagen I alpha 1 standard). The plate was incubated at room temperature for 2 h. After washing, 100 µL of the Pro-Collagen I alpha 1 Detection Antibody was added. The plate was incubated for another 2 h. Following washes, 100 µL of Streptavidin-HRP (R&D Systems, Minneapolis, MN, USA) solution was added and the plate was incubated for 20 min. Next, 100 µL of the HRP substrate solution was added and incubated for an additional 20 min. Finally, 50 µL of the stop solution was added to each well and the absorbance was measured by a microplate reader (INNO-M, LTEK, Seongnam, Republic of Korea) at 450 nm.

### 2.5. In Vivo Animal Study

An in vivo study was conducted using hairless mice to evaluate the efficacy of collagen synthesis. Seven-week-old female SKH-1/60 hairless mice were purchased from Orient Bio (Seongnam, Republic of Korea) and housed with unrestricted access to food and water throughout the course of the study. The study protocol was approved by the Institutional Committee for Ethics in Animal Research of CHA Medical University (Approval no. IACUC-230131) and adhered to the Animal Protection Act and Guidelines for the Care and Use of Laboratory Animals. A total of eight mice for the groups were used for each time point. Injections of 250 µL of mPEG-PDLLA copolymers (10% aqueous solution) and Rejuran^®^ were administered into two distinct areas (left and right) of the dorsal sites, respectively. For each time point (1 day, 1 week, 4 weeks, 8 weeks, 12 weeks, 16 weeks, and 24 weeks post-injection), skin tissues from eight mice were collected to assess gene expression and observe angiogenesis, as well as for hematoxylin and eosin (H&E) and Masson’s trichrome staining.

#### 2.5.1. Quantitative Real-Time Polymerase Chain Reaction

Using the Accu-Power^®^ GreenStar^TM^ qPCR premix (Bioneer, Daejeon, Republic of Korea) in combination with the Exicycler^TM^ apparatus (Bioneer, Daejeon, Republic of Korea), the expression levels of target genes were assessed by qRT-PCR. Initial denaturation at 96 °C for 5 min, 40 cycles of denaturation at 96 °C for 30 s, annealing at 60 °C for 30 s, and extension at 72 °C for 30 s comprised the PCR conditions. Comparative CT (ΔΔCT) was used to examine gene expression levels.

#### 2.5.2. Paraffin-Embedded Block Preparation and Measurement of Dermal Thickness

Samples of skin tissue were preserved in cold 4% paraformaldehyde (Sigma-Aldrich, St. Louis, MO, USA) and then rinsed with PBS for half an hour. The fixed tissues were cleaned in xylene, embedded in paraffin, and dehydrated using an escalating ethanol gradient (70, 80, and 90%). Blocks of paraffin-embedded tissue were cut with a microtome (Thermo Fisher Scientific, Rockford, IL, USA) into slices that were 5 µm thick. To improve tissue adhesion, the pieces were air-dried and roasted for a whole night at 65 °C. H&E staining and Masson’s trichrome staining images were captured using a slide scanner (Carl Zeiss, Axio Scan.Z1, Oberkochen, Germany), and the length of the dermal layer was measured using ImageJ software (NIH, Bethesda, MD, USA). The thickness of the dermal was the average length measured at four different randomly selected parts for the tissue section of each sample.

#### 2.5.3. Statistical Analysis

Statistical comparisons between groups were performed using one-way ANOVA. The obtained results were tested by Turkey’s multiple comparisons test and a *p*-value of less than 0.05 was considered statistically significant.

## 3. Results and Discussion

### 3.1. Characterization of mPEG-PDLLA Copolymers

mPEG-PDLLA copolymers of different molecular weights were synthesized to evaluate their physicochemical properties, including solubility, particle size, and yield (Table 1). Poly(D,L-lactic acid) (PDLLA) is PEGylated with methoxy-polyethylene glycol (mPEG) to enhance its solubility and biocompatibility, making it easier to administer in microsphere formulations. Three copolymers were prepared and designated as P1, P2, and P3. The number-average molecular weight (M_n_) of P1 was found to be 5360, the M_w_ was 6322 and the polydispersity index (PDI) was 1.17. It was also tested for solubility in distilled water and found to be stably dispersed. For P2, the M_n_ was 6603, the M_w_ was 8496, and the PDI was 1.28. It was also observed to be stable in the liquid phase. In contrast, P3 exhibited an M_n_ of 8385, an M_w_ of 12,881, and a PDI of 1.53. Due to its high molecular weight, P3 was found to precipitate in the liquid phase, indicating insolubility under the test conditions. The new PEGylated PDLLA copolymer has a significantly lower molecular weight (6000~8500 Da) compared to PLLA-based products (~80,000 Da) [33], and it is expected to degrade more quickly in vivo. This can be advantageous for controlled and predictable biodegradation, potentially leading to a shorter but more controlled stimulation of collagen production. Moreover, the production yield of P2 (87.5%) was observed to be higher than that of P1 (78.1%), demonstrating the superior efficiency of P2. Based on the results, P2 was selected for further study.

**Figure 2:**
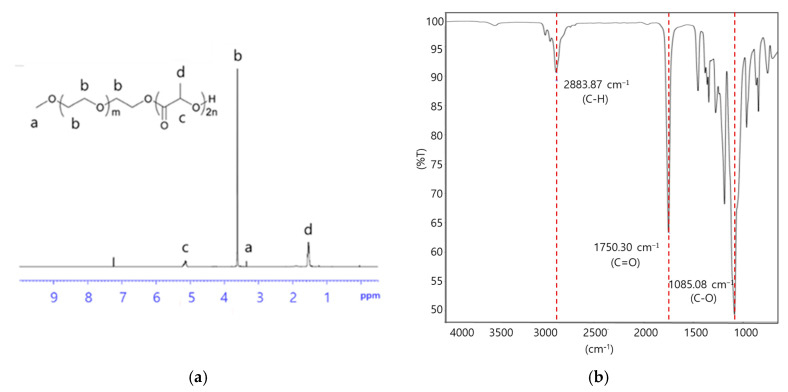
(**a**) ^1^H-NMR spectrum and (**b**) FT-IR spectrum of mPEG-PDLLA copolymers.

The chemical structure of the synthesized mPEG-PDLLA was further confirmed by the ^1^H-NMR spectrum in Figure 2a. Peaks observed at 3.40 ppm (Figure 2a, (a)) and 3.65 ppm (Figure 2a, (b)) were assigned to the methyl and methylene protons of the mPEG segment, respectively [34]. Similarly, peaks at 1.50 ppm (Figure 2a, (d)) and 5.20 ppm (Figure 2a, (c)) corresponded to the methyl and methylene within the PDLLA segment. A proton signal at 4.35 ppm was attributed to the methylene group adjacent to the PDLLA segment.

FT-IR analysis provided additional evidence of the successful synthesis of the mPEG-PDLLA in Figure 2b. The characteristic peak for mPEG was observed at 2883.87 cm^−1^, representing the stretching vibration of the C-H group. Peaks at 1750.30 and 1085 cm^−1^ were attributed to the C=O and C-O groups of D,L-lactide, respectively [35]. These combined spectroscopic results confirmed the successful synthesis of mPEG-PDLLA copolymers.

**Figure 3:**
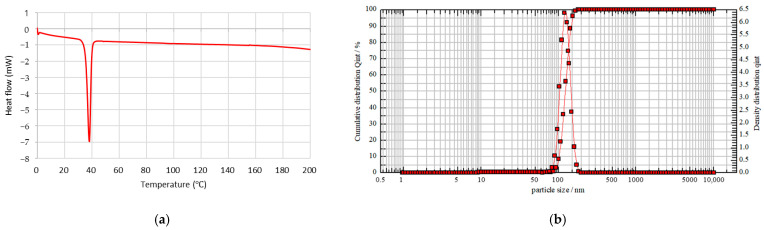
(**a**) Thermodynamic property of mPEG-PDLLA by DSC analysis. (**b**) Size distribution of mPEG-PDLLA by particle size analyzer.

Thermal analysis of the mPEG-PDLLA copolymers was evaluated using DSC. A single heating ramp was applied over a temperature range of 0 to 200 °C and the results are depicted in Figure 3a.

DSC analysis was conducted to examine the crystallinity of mPEG-PDLLA, as PDLLA is generally known to have an amorphous structure and is incapable of crystallization. As shown in Figure 3a, mPEG-PDLLA exhibited an endothermic melting peak around 38 °C, attributed to the crystallization of mPEG. Since PDLLA is amorphous, it does not exhibit a melting temperature (T_m_), but only a glass transition temperature (T_g_). Sedush et al. reported that for Sculptra^®^ and Gana V^®^, which are primarily composed of PLLA (a crystalline polymer), endothermic melting peaks attributed to PLLA were observed at 120~180 °C. In contrast, for AestheFill^®^ and Repart PLA^®^, which are based on PDLLA (an amorphous polymer), no endothermic melting peaks were detected [33]. The T_g_ of low-molecular-weight PDLLA is typically around 40~45 °C. In general, higher molecular weight results in a slight increase in T_g_ and T_m_. The thermal properties of polymers are closely related to the mobility of polymer chains [36]. According to the DSC data, the T_g_ of PDLLA decreased to below 40 °C, resulting in the absence of a positive exothermic peak. Instead, a negative exothermic peak was observed at a similar temperature, opposite to that of mPEG. In addition, a previous study has reported that PLA (Sculptra^®^) exhibits a melting peak at approximately 170 °C [33]. However, in this study, the absence of a peak within this temperature range confirms the successful synthesis of PDLLA.

The hydrodynamic size of mPEG-PDLLA copolymers was determined using a nanoparticle size analyzer, as shown in Figure 3b. The mPEG-PDLLA showed a unimodal size distribution with an average particle size of 121 nm obtained. In addition, the PDI was observed to be 0.027. This PEGylation helps reduce side effects such as edema, nodules, and hemorrhagic spots, which are common concerns with existing poly-L-lactic acid (PLLA) microsphere-based products like Sculptra^®^.

### 3.2. In Vitro Cytotoxicity and Collagen Synthesis Assay

**Figure 4:**
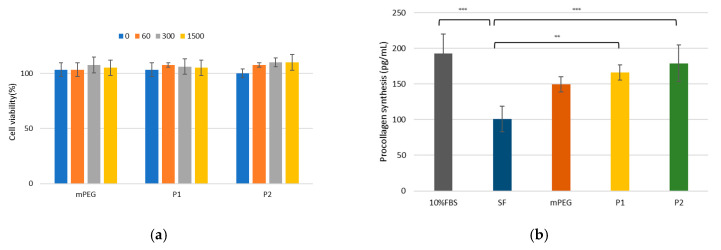
(**a**) Cell viability in HDF cells. HDF cells were treated with varying concentrations (0, 60, 300, and 1500 ppm) for 1 day. (**b**) Collagen synthesis assay for mPEG-PDLLA copolymers in HDF cells treated at 300 ppm concentration for 1 day. (** *p* < 0.01, and *** *p* < 0.001, *n* = 3), (SF: serum-free, P1: mPEG-PDLLA (M_w_: 6322 g/mol), and P2: mPEG-PDLLA (M_w_: 8496 g/mol)).

The cytotoxicity of mPEG-PDLLA was evaluated using a cell viability assay with HDF cells at various concentrations (0, 60, 300, and 1500 ppm). As shown in Figure 4a, cell viability consistently remained above 100%, even at the highest concentration of 1500 ppm, demonstrating that the mPEG-PDLLA exhibited negligible cytotoxicity. Furthermore, no significant differences in cell viability were observed between mPEG and mPEG-PDLLA copolymers, suggesting that the addition of the PDLLA segment did not induce any cytotoxic effects. This result highlights the biocompatibility and safety of the mPEG-PDLLA.

To assess the potential of mPEG-PDLLA in promoting collagen synthesis, a collagen synthesis assay was performed. In this assay, 10% FBS was used as a positive control, while serum-free (SF) medium served as the negative control. As anticipated, 10% FBS exhibited the highest collagen synthesis capability among all tested samples, while the negative control demonstrated the lowest performance, as shown in Figure 4b. The mPEG-PDLLA exhibited a significant ability to enhance collagen synthesis compared to the negative control, indicating their potential utility as a regenerative material. To further validate these findings, we performed an ANOVA, followed by Tukey’s multiple comparisons test. The results showed statistically significant differences between 10% FBS and SF, SF and P1, and SF and P2 (*p* < 0.01), whereas no significant differences were observed among the other groups (*p* > 0.05).

In general, PEGylation is known to reduce protein adsorption and immune recognition; however, other studies have reported that PEG-based materials can still interact with the immune system under certain conditions [29,37]. For this reason, mPEG also demonstrated a certain level of collagen regeneration capability in the in vitro test. mPEG-PDLLA has also been shown to stimulate fibroblast activation and initiate an inflammatory response, which leads to increased collagen production over time. As mPEG-PDLLA degrades, it releases lactic acid, which further accelerates the breakdown of PDLLA. This process lowers the local pH, creating an environment that fosters fibroblast proliferation and sustained collagen synthesis. However, the immediate effect of lactic acid is considered minimal in in vitro tests, as these assessments are evaluated at a specific time point. Nevertheless, the improved collagen regeneration capabilities of mPEG-PDLLA are promising compared to mPEG. The localized acidic pH environment from lactic acid degradation may accelerate the breakdown of PDLLA and stimulate fibroblast activation, further enhancing its collagen regeneration capability; as a result, in the long term, the difference in collagen regeneration between the two groups is expected to become more distinct.

### 3.3. Comparative Gene Expression In Vivo

#### 3.3.1. Angiogenesis and Inflammation in Skin Rejuvenation

Throughout the animal study, no adverse symptoms, inflammations, or deaths were observed. Additionally, there was no significant difference in the average weight between groups. Irritation scores also confirmed that all mice remained “symptom-free” during the 24 weeks following the injection of the test substances.

**Figure 5:**
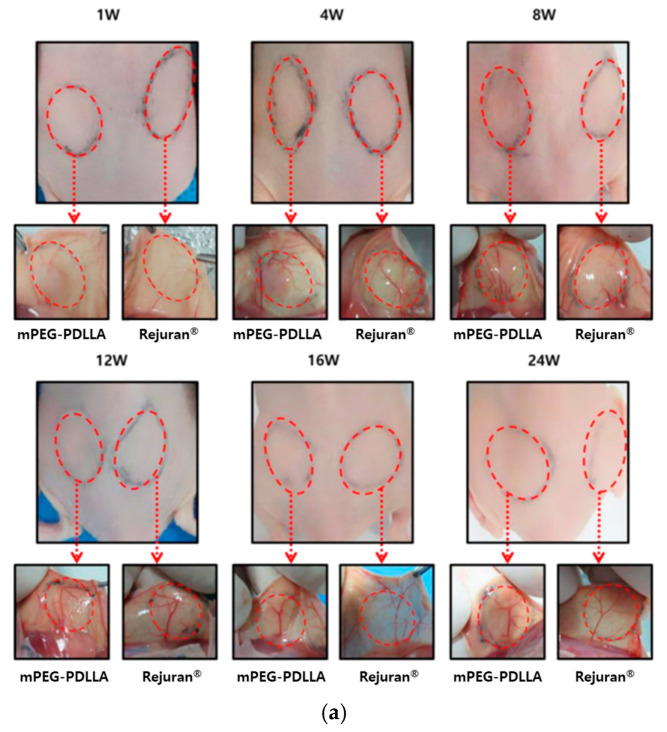

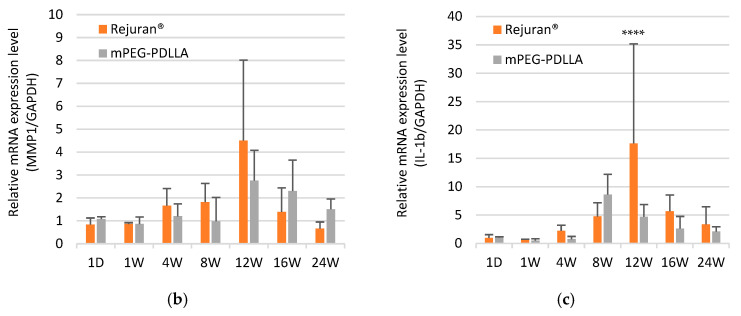
(**a**) Post-modern examination results following subcutaneous injections of Rejuran^®^ and mPEG-PDLLA copolymers. Comparison of gene expression for Rejuran^®^ and mPEG-PDLLA copolymers. (**b**) MMP1 and (**c**) IL-1b. **** *p* < 0.001 vs. Rejuran^®^ (*n* = 5/group).

As shown in Figure 5a, the angiogenesis of hairless mice injected with 250 μL each of Rejuran^®^ and mPEG-PDLLA at the dorsal site was observed over time to assess the collagen regeneration effect induced by the inflammatory response. In the early phase of the injection (1~8 W), both groups exhibited neovascularisation, though this was more evident in the mPEG-PDLLA group. In addition, in the middle period of observation (12 W~16 W), Rejuran^®^’s blood vessels became irregular or disappeared; however, mPEG-PDLLA maintained a relatively intact vasculature. This indicates that the inflammatory response is not excessive and that tissue regeneration is balanced. Finally, at the end of the observation (24 W), Rejuran^®^’s blood vessels faded, which can be interpreted as a decline in vascular stability due to the excessive inflammatory response that occurred after 12 weeks. In contrast, mPEG-PDLLA has been demonstrated to promote prolonged retention of blood vessels, suggesting its capacity to facilitate long-term skin rejuvenation.

In order to determine an objective assessment of the ability of the inflammatory response to regenerate collagen, gene expression analysis was performed with the results of Matrix Metalloroteinase-1 (MMP1) and Interleukin-1 beta (IL-1b) (Figure 5b,c). MMP1 is an enzyme involved in the breakdown of collagen and the promotion of tissue remodeling, and plays a role in the process of angiogenesis by reorganizing existing tissue and aiding cell migration. However, when MMP1 is overexpressed, it has been demonstrated to cause excessive collagen breakdown, resulting in a weakening of the skin’s structure. As demonstrated in Figure 5b, Rejuran^®^ exhibited a substantial augmentation in the expression of MMP1 at 12 weeks. In contrast, the expression levels of mPEG-PDLLA were found to be comparable to or lower than those of Rejuran^®^. Relatively overexpressed MMP1 in Rejuran^®^ is likely to have promoted collagen degradation, weakening the skin structure, which supports the neovascularization results shown in Figure 5a. Figure 5c shows a comparison of the expression of IL-1b. IL-1b, a cytokine, has been demonstrated to induce an inflammatory response. Overexpression of IL-1b has been shown to create an excessive inflammatory environment. Although adequate levels of IL-b1 are known to be beneficial for angiogenesis, an excessive inflammatory response can be induced by a sharp increase, which was observed in Rejuran^®^’s results at 12 weeks. This excessive response can damage vascular stability.

#### 3.3.2. Gene Expression in Collagen Regeneration

**Figure 6:**
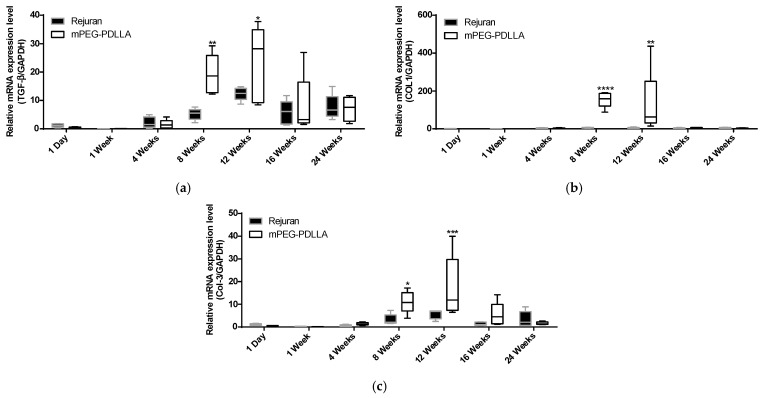
Comparison of gene expression for Rejuran^®^ and mPEG-PDLLA copolymers. (**a**) TGF-β, (**b**) COL1, and (**c**) COL3. * *p* < 0.05, ** *p* < 0.01, *** *p* < 0.005, and **** *p* < 0.001 vs. Rejuran^®^ (*n* = 5/group).

Three genes were evaluated: collagen type I (COL1), collagen type III (COL3), and transforming growth factor-β (TGF-β). TGF-β has long been considered to be an important mediator for tissue repair and fibrosis [38]. The expression of TGF-β genes showed a significant increase in the mPEG-PDLLA at 8 and 12 weeks compared to the Rejuran^®^ in Figure 6a. Rejuran^®^ demonstrated a statistically significant increase at 12 weeks, whereas mPEG-PDLLA exhibited a statistically significant increase at 8 weeks. Therefore, expression of the TGF-β gene was observed 4 weeks earlier in mPEG-PDLLA compared to Rejuran^®^. Notably, gene expression in the mPEG-PDLLA was maintained for up to 12 weeks.

COL1 is the most abundant collagen, a fibrous protein widely distributed in the skin, tendons, ligaments, blood vessels, bones, lungs, and heart. Specifically, COL1 is a principal structural component of the dermis and is crucial for maintaining the strength and integrity of tissue [39]. COL3 plays a pivotal role in tissue repair by promoting regenerative healing and minimizing scar formation. It also contributes significantly to bone development and fracture repair by influencing stem cell function and angiogenesis. A deficiency in COL3 is associated with impaired healing, increased scar formation, and skeletal abnormalities [40]. TGF-β is widely recognized for its potential to induce COL3 mRNA expression, as evidenced by the observation of a similar gene expression pattern [41].

For COL1, the mPEG-PDLLA exhibited a notable elevation in gene expression at 8 weeks compared to the Rejuran^®^ (Figure 6b). Regarding COL3, the mPEG-PDLLA exhibited a significant 3.4-fold increase in expression at 8, 12, and 16 weeks compared to Rejuran^®^ in Figure 6c. In contrast, Rejuran^®^ did not show any significant increase in COL3 expression.

### 3.4. Dermal Thickness by H&E Staining in Animal Study

**Figure 7:**
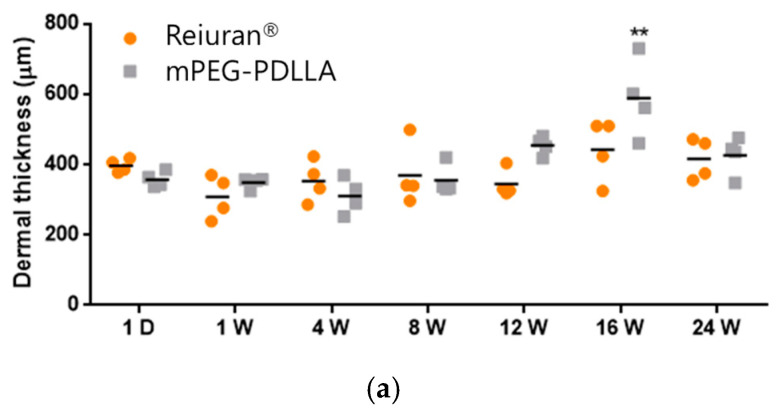

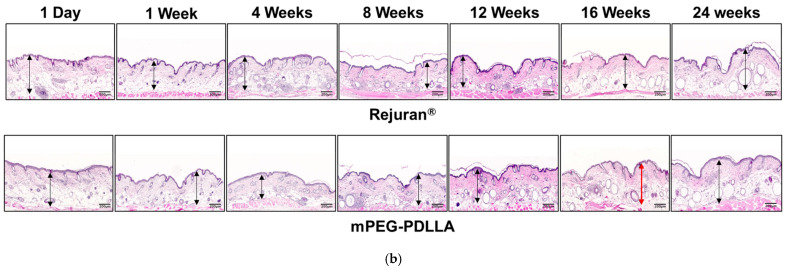
H&E staining results for Rejuran^®^ and mPEG-PDLLA copolymers: (**a**) sum and standard deviation of measured dermal thickness. The mPEG-PDLLA exhibited a progressive increase in dermal thickness in select tissues starting from 12 weeks, which became statistically significant by 16 weeks. (** *p* < 0.01 vs. Rejuran^®^) (*n* = 4/group). (**b**) Representative images of H&E-stained tissues. Dermal thickness increased significantly in the mPEG-PDLLA at 16 weeks (*p*-value = 0.0087).

H&E staining was conducted to assess cell density, collagen fiber structure, inflammatory responses, and the quality of regenerated tissue. In this study, Rejuran^®^ and mPEG-PDLLA were injected to evaluate their effects on collagen regeneration at the tissue level and to monitor long-term in vivo responses in Figure 7. During the first week post-injection, both groups exhibited mild inflammatory responses and an increase in fibrous tissue, with no statistically significant differences observed between them. However, over time, the mPEG-PDLLA demonstrated a greater increase in dermal thickness, which reached its peak at 16 weeks (Figure 7b, red arrow), showing a statistically significant difference compared to the Rejuran^®^. At 24 weeks post-injection, a reduction in tissue density was observed in the Rejuran^®^, while the mPEG-PDLLA exhibited relatively stable tissue density. This observation suggests that the rapid degradation rate of Rejuran^®^ may limit its long-term efficacy in promoting collagen regeneration. In addition, the increased MMP1 and IL-1b from 12 weeks onwards promoted collagen degradation and resulted in an excessive inflammatory response, which may have contributed to the structural instability of neovascularization and collagen. In contrast, the mPEG-PDLLA, with its gradual degradation profile, is anticipated to support sustained collagen regeneration over an extended period.

### 3.5. Collagen Genesis by Masson’s Trichrome Staining

**Figure 8:**
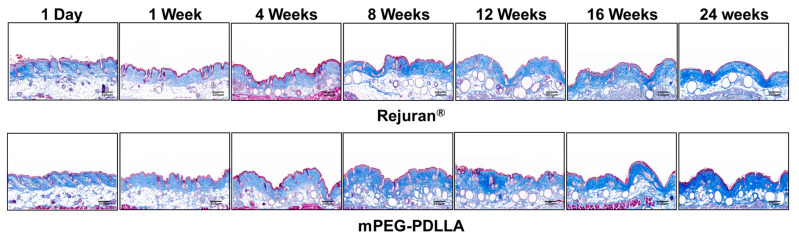
Masson’s trichrome staining result for Rejuran^®^ and mPEG-PDLLA (blue: collagen, red: muscle fibers, keratine, and cytoplasm). In the Rejuran^®^, collagen fiber density was increased in select tissues from 16 weeks onward. Conversely, in the mPEG-PDLLA, collagen fiber density increased as early as 12 weeks.

Masson’s trichrome staining was employed to specifically highlight collagen within connective tissues, in contrast to H&E staining. In this staining method, collagen is visualized as blue, muscle or cytoplasm as red, and cell nucleus as black in Figure 8. The analysis revealed that up to the first week post-injection, both the Rejuran^®^ and mPEG-PDLLA showed limited collagen formation, with no statistically significant differences between the two groups. However, from 4 weeks onward, the mPEG-PDLLA demonstrated a distinct and progressively thicker collagen layer, forming denser and more organized collagen over time. By 16 weeks, the collagen layer reached its maximum density and the tissue structure appeared stable. At 24 weeks, the collagen layer remained well defined, aligning with the findings observed in the H&E staining results. In contrast, the Rejuran^®^ exhibited a lower-density collagen layer as early as 8 weeks, which was significantly less pronounced than that observed in the mPEG-PDLLA. This trend persisted, with reduced collagen regeneration in the Rejuran^®^ compared to the mPEG-PDLLA after 16 weeks and beyond.

Shi et al. suggested that PEG, a hydrophilic polymer, is widely recognized for its ability to evade uptake by the mononuclear phagocytic system (MPS), thereby extending the circulation time of nanoparticles within the body—a phenomenon commonly referred to as the “stealth effect” [42]. It was cited as being less susceptible to macrophage activity during IV infusion and therefore likely to be viable in terms of biodegradation. Although PEGylation is known to reduce protein adsorption and immune recognition, some studies suggest that PEG-based materials can still interact with the immune system under certain conditions. Macrophages play a key role in stimulating fibroblast activation, which is crucial for tissue remodeling. When mPEG-PDLLA is injected into the dermis or below the subcutaneous layer of skin, it induces gene expression and activates various signaling pathways, leading to angiogenesis and the formation of new blood vessels. Subsequently, the COL3 gene stimulates the expression of the TGF-β gene, gradually promoting collagen regeneration. It is presumed that the mPEG-PDLLA, the micelle-like structure formed by the mPEG coating around the PDLLA core, is hypothesized to degrade slowly.

## 4. Conclusions

This study identified optimal conditions for using mPEG-PDLLA copolymers as a skin booster and discussed their synthesis and physicochemical properties. Poly D,L-lactic acid was able to dissolve the hydrophobic polymer easily due to the PEGylation with hydrophilic polyethylene glycol (PEG) compared to conventional poly-lactic acid microspheres. This approach is chosen because nanoparticles, due to their small size, can be more easily injected into the sub-dermis. The mPEG-PDLLA exhibited an average size of around 120 nm and a unimodal size distribution in the aqueous phase. The physicochemical properties of the mPEG-PDLLA were also confirmed by the NMR spectrum, with peaks of 3.40 and 3.65 ppm for mPEG, and 1.50 and 5.20 ppm for PDLLA. Furthermore, the FT-IR spectrum was used to identify the characteristic peaks of mPEG and D,L-lactide identified in 2883, 1750, and 1085 cm^−1^. DSC analysis confirmed the thermal properties of the mPEG-PDLLA.

In vitro cytotoxicity tests and biocompatibility evaluations verified that mPEG-PDLLA is non-cytotoxic, with no negative side effects observed at the tested doses. Furthermore, in the in vivo study, mPEG-PDLLA exhibited superior gene expression and collagen regeneration capabilities compared to Rejuran^®^. Our results from animal experiments indicated that the material did not show any toxicity or tissue infiltration. Although not included in this study, the biological safety evaluation results were satisfactory. These findings highlight their potential as a safe and effective biomaterial for use in cosmetic and regenerative aesthetic formulations. Therefore, mPEG-PDLLA copolymers show significant potential as a dermal filler in skin rejuvenation.

## 5. Patent

Application no. 10-2023-0018852 (Republic of Korea).

## Figures and Tables

**Table 1:** The synthesized mPEG-PDLLA copolymers in this study.

Code	M_n_ (g/mol) ^a^	M_w_ (g/mol) ^b^	PDI ^c^	Yield (%) ^d^	Solubility ^e^
P1	5360	6322	1.17	78.1	Soluble
P2	6603	8496	1.28	87.5	Soluble
P3	8385	12,881	1.53	84.4	Insoluble

^a^ Number-average molecular weight determined by GPC. ^b^ Weight-average molecular weight determined by GPC. ^c^ Polydispersity index determined by GPC (PDI = M_w_/M_n_). ^d^ Yield of mPEG-PDLLA copolymers. ^e^ Samples were prepared at a concentration of 10 wt/v%.

## Data Availability

The data presented in this study are available upon request from the corresponding author.
